# Altered Cerebellar White Matter in Sensory Processing Dysfunction Is Associated With Impaired Multisensory Integration and Attention

**DOI:** 10.3389/fpsyg.2020.618436

**Published:** 2021-02-03

**Authors:** Anisha Narayan, Mikaela A. Rowe, Eva M. Palacios, Jamie Wren-Jarvis, Ioanna Bourla, Molly Gerdes, Annie Brandes-Aitken, Shivani S. Desai, Elysa J. Marco, Pratik Mukherjee

**Affiliations:** ^1^Department of Radiology and Biomedical Imaging, University of California, San Francisco, San Francisco, CA, United States; ^2^Department of Medicine, Tulane University School of Medicine, New Orleans, LA, United States; ^3^Cortica Healthcare, San Rafael, CA, United States; ^4^Department of Neurology, University of California, San Francisco, San Francisco, CA, United States; ^5^Department of Bioengineering and Therapeutic Sciences, University of California, San Francisco, San Francisco, CA, United States

**Keywords:** sensory processing, cerebellum, neurodevelopmental disorders, diffusion tensor imaging, connectivity, white matter, fractional anisotropy

## Abstract

Sensory processing dysfunction (SPD) is characterized by a behaviorally observed difference in the response to sensory information from the environment. While the cerebellum is involved in normal sensory processing, it has not yet been examined in SPD. Diffusion tensor imaging scans of children with SPD (*n* = 42) and typically developing controls (TDC; *n* = 39) were compared for fractional anisotropy (FA), mean diffusivity (MD), radial diffusivity (RD), and axial diffusivity (AD) across the following cerebellar tracts: the middle cerebellar peduncles (MCP), superior cerebellar peduncles (SCP), and cerebral peduncles (CP). Compared to TDC, children with SPD show reduced microstructural integrity of the SCP and MCP, characterized by reduced FA and increased MD and RD, which correlates with abnormal auditory behavior, multisensory integration, and attention, but not tactile behavior or direct measures of auditory discrimination. In contradistinction, decreased CP microstructural integrity in SPD correlates with abnormal tactile and auditory behavior and direct measures of auditory discrimination, but not multisensory integration or attention. Hence, altered cerebellar white matter organization is associated with complex sensory behavior and attention in SPD, which prompts further consideration of diagnostic measures and treatments to better serve affected individuals.

## Introduction

Sensory processing dysfunction (SPD), also referred to as sensory integration disorder and sensory processing disorder, is a behaviorally described neurodevelopmental difference estimated to affect up to 16% of children in the general population, and 40–80% of children with other comorbid neurodevelopmental disorders, such as autism spectrum disorder (ASD) or attention deficit hyperactivity disorder (ADHD; Ahn et al., 2004; Koziol et al., 2011). This increasingly recognized neurodevelopmental disorder affects the brain's capacity to respond and adapt to a continuous flow of unimodal and/or multimodal sensory stimuli and significantly compromises learning and daily functioning (Miller et al., 2007; Miller, 2009; Ryckman et al., 2017). Despite verbal and other intellectual performance abilities in the normal range, individuals with SPD can experience substantial difficulty with fine motor control, cognitive control, and behavioral regulation (Brandes-Aitken et al., 2018), leading to emotional and social difficulties including anxiety and negative self-image (Miller et al., 2007; Miller, 2009; McMahon et al., 2019).

While SPD refers to an umbrella of phenotypic challenges (Miller et al., 2007), the most recognizable aspects of the condition are sensory over-responsivity, or hyperreactivity, and sensory under-responsivity, or hyporeactivity. The DSM-5 does not recognize SPD as a standalone disorder, though sensory hyper- and hypo-reactivity are listed as core symptoms of ASD (American Psychiatric Association., 2013). Despite this overlap of SPD features and ASD, SPD also occurs in isolation in individuals who do not have the primary social communication challenges that form the ASD criteria (Ahn et al., 2004; Tavassoli et al., 2018). A 2018 study demonstrated that children who have SPD without ASD demonstrate higher empathy scores and less systematizing behavior than those with ASD (Tavassoli et al., 2018). In addition to behavioral features, SPD and ASD also differ with regard to certain neuroanatomical markers, including differences in white matter (WM) microstructural integrity of neural tracts that subserve socio-emotional processing (Chang et al., 2014). The findings from these previous studies provide evidence that atypical sensory reactivity is not simply a feature of the broader autism construct. However, our understanding of cerebellar differences in children with atypical neurodevelopment largely stems from the study of individuals with ASD.

Previous studies have demonstrated cerebellar differences between ASD and typically developing controls (TDC) at the cellular, structural, and functional level (Kern, 2002; Koziol et al., 2011). Several diffusion tensor imaging (DTI) studies of ASD have shown alterations of WM microstructure in the pathways linking the cerebellum to the brainstem and cerebral hemispheres (Brito et al., 2009; Shukla et al., 2010; Groen et al., 2011; Jeong et al., 2012). A 2017 structural MRI study of individuals with ASD showed differences in morphological connectivity among neuroanatomical areas implicated in sensory processing, including sensory regions of the cerebral cortex, the amygdala, and the cerebellum, with altered connectivity between the cerebellum and cortical areas (Cardon et al., 2017). Moreover, the cerebellum plays a role in multisensory processing, as its connectivity to the cerebral cortex is important for sensory integration across different modalities such as hearing, sight, touch and even smell (Cardon et al., 2017; Zhao et al., 2018; Sathyanesan et al., 2019). While investigation of the structural and functional aspects of sensory processing has begun in children with isolated SPD and those with sensory over-responsivity and autism (Bar-Shalita et al., 2009; Marco et al., 2011; Brandes-Aitken et al., 2018; Molholm et al., 2020), there has so far been no exploration of the role of the cerebellum in children with isolated SPD. We posit that the cerebellum may contribute to acquisition, discrimination, modulation, and integration of multisensory information for interpretation of the environment and generation of appropriate responses.

DTI is a neuroimaging method that measures the diffusion of water in the brain's white matter. It allows us to visualize and quantify direction, uniformity, and orientation of WM tracts for the examination of structural connectivity, e.g., between cerebellum and sensory cortex areas in ASD (Jeong et al., 2012; Cardon et al., 2017; Mallott et al., 2019). Thus, DTI measures can serve as a proxy for connectivity, especially in studying distributed neural networks, and can reveal subtle microstructural anomalies undetectable by other non-invasive imaging methods (Payabvash et al., 2019). Commonly used DTI metrics include fractional anisotropy (FA), mean diffusivity (MD), radial diffusivity (RD), and axial diffusivity (AD; Alexander et al., 2007). In typical WM maturation, there are increases in FA, and decreases in MD and RD, with relatively less change in AD (Mukherjee and McKinstry, 2006). Although the underlying neurobiological processes are not completely understood, this is likely because myelination and other aspects of WM maturation reduce water diffusion preferentially perpendicular to the fibers rather than parallel to the fibers. Correspondingly, our previous DTI studies of SPD have shown decreases in FA and increases in MD and RD in the WM of the cerebral hemispheres, which we collectively refer to as “decreased microstructural integrity” (Owen et al., 2013; Chang et al., 2014, 2016). Although prior studies have examined sensory processing using electroencephalography (EEG), magnetoencephalography (MEG), MRI, and functional MRI (fMRI), none to our knowledge have used DTI to study the cerebellum or brainstem of children with SPD.

**Figure 1:**
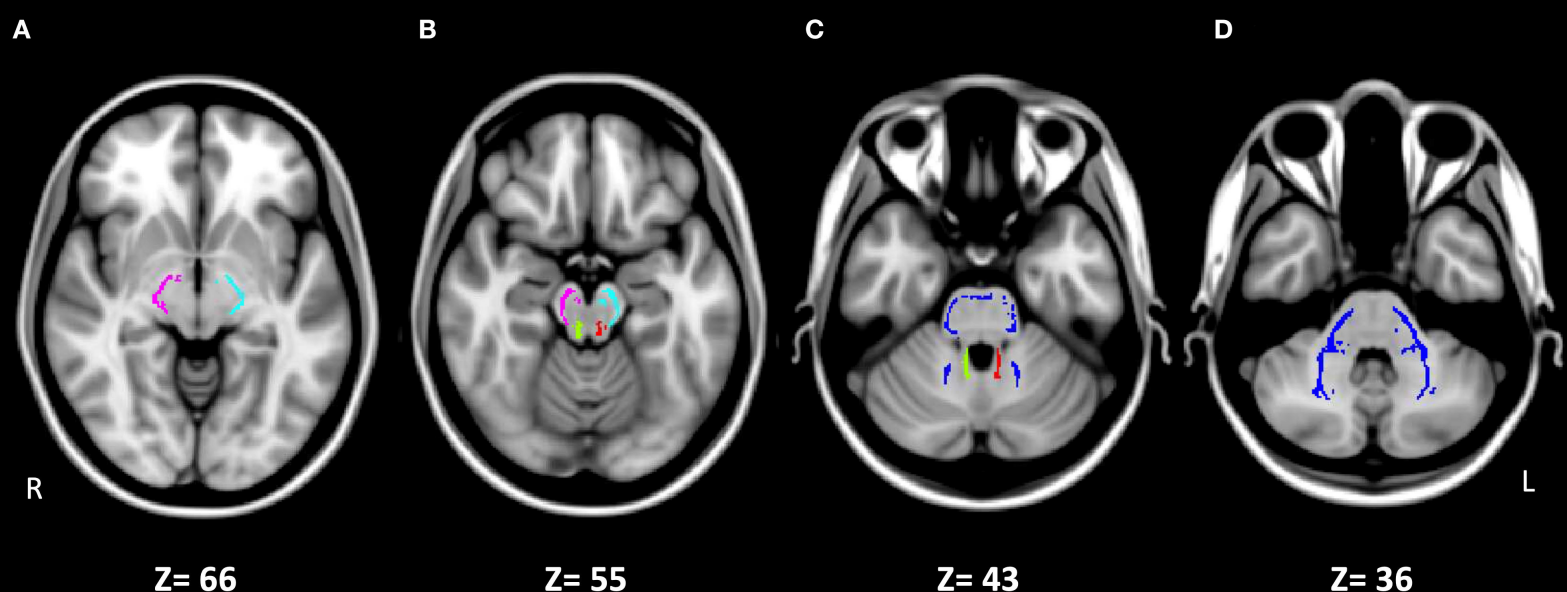
Johns Hopkins University ICBM-DTI-81 White-Matter Labeled Atlas regions of interest on the white matter skeleton in MNI152 space for the five studied tracts. **(A)** Right cerebral peduncle (CP-R, pink) and left cerebral peduncle (CP-L, light blue). **(B)** CP-R (pink), CP-L (light blue), right superior cerebellar peduncle (SCP-R, green), and left superior cerebellar peduncle (SCP-L, red). **(C)** Middle cerebellar peduncle (MCP, dark blue), SCP-R (green), and SCP-L (red). **(D)** MCP (dark blue). Images are presented in radiological convention (left hemisphere on right side of image).

In this study, we use DTI to investigate the major output tracts of the cerebellum, the left and right superior cerebellar peduncles (SCP-L and SCP-R), as well as the major input tract to the cerebellum, the middle cerebellar peduncle (MCP), at its decussation. We also investigate two control WM regions in the brainstem, the left and right cerebral peduncles (CP-L and CP-R) at the midbrain, which contain cerebral fiber tracts that connect to subcortical structures in the lower brainstem and spinal cord (Figure 1). We hypothesize that the CP, SCP, and MCP will all show decreased microstructural integrity, characterized by an increase of RD and, to a lesser extent, of MD, as well as a decrease of FA, which we have previously demonstrated for cerebral WM tracts in SPD compared to typically developing children (Owen et al., 2013; Chang et al., 2016).

However, we postulate a dissociation in the relationship of WM microstructure in the cerebrum vs. cerebellum with sensory processing and behavior. We hypothesize that the CP will most strongly correlate with unimodal sensory processing measures such as the Differential Screening Test for Processing (DSTP) and the Auditory and Tactile measures of the Sensory Profile, consistent with prior results from cerebral tracts in SPD (Owen et al., 2013; Chang et al., 2016). In contradistinction, because of the integral role of the cerebellum in multisensory integration (Cardon et al., 2017; Zhao et al., 2018; Sathyanesan et al., 2019) and in attention (Buckner, 2013; Baumann et al., 2015; Ailion et al., 2020), we hypothesize that the SCP and MCP will correlate more strongly than does the CP with measures of Multisensory Processing and Attentional Behavior from the Sensory Profile. Furthermore, we expect these correlations of cerebellar WM microstructure with sensory behavior to exist in the SPD cohort, but not in TDC who by definition do not exhibit atypical sensory processing or cognitive control.

## Materials and Methods

### Study Design

Children 8 to 12 years of age were enrolled in this study from the University of California, San Francisco (UCSF) Sensory Neurodevelopment and Autism Program phenotype database (pediLAVA) and neuroimaging collection. The study design was approved by the UCSF Institutional Review Board and informed consents and assents were obtained from primary caregivers and study participants, respectively, in accordance with IRB policy. Consistent with previous studies in our lab, research designation of SPD was determined using the long-form Sensory Profile (SP; Dunn, 1999), a parent report measure consisting of 125 questions about sensory behaviors with a 5-point Likert scale response. Totals in each sensory domain are then categorized into “Typical Performance,” “Probable Difference,” or “Definite Difference.” On the SP, higher scores are indicative of “Typical Performance,” while lower scores indicate increased sensory symptomatology, or a “Definite Difference.” SPD designation required a score in the “Definite Difference” range for at least one of the following sections of the SP: Auditory Processing, Visual Processing, Vestibular Processing, Tactile Processing, Multisensory Processing, or Oral Sensory Processing. To ensure all cases of SPD were recognized, a Short Sensory Profile (SSP) total score was calculated by adding the responses to the subset of 38 questions that make up the SSP. Those with a SSP total score in the “Definite Difference” range were also included in the SPD cohort. To be included in the TDC group, individuals could not meet any of the above criteria for a research diagnosis of SPD. The Social Communication Questionnaire (SCQ; Rutter et al., 2003) parent report form was administered to all subjects to screen for ASD. Those scoring at or above the cutoff of 15 on the SCQ were then evaluated using the Autism Diagnostic Observation Schedule (ADOS; Lord et al., 1989). Individuals scoring above the ADOS diagnostic cutoff were excluded from analysis. Additional exclusion criteria included premature delivery (<37 weeks), brain malformation or injury, presence of a known genetic diagnosis (e.g., Fragile X syndrome), movement disorder, bipolar disorder, psychotic disorder, or hearing impairment, which were screened for during the intake process. ADHD was not formally assessed, though most participants (SPD: *n* = 35; TDC: *n* = 32) completed either the parent-report Child Symptom Inventory or the Vanderbilt ADHD Diagnostic Rating Scale (Gadow and Sprafkin, 2002; Wolraich et al., 2003). None of the TDC screened positive for ADHD, while 17 of the 35 participants with SPD screened for ADHD did. Participants in the SPD group were not excluded for ADHD symptoms.

### Behavioral Measures

In addition to informing research diagnostic criteria, the SP yields useful information about individual sensory domains and sensory-associated responses and behaviors. In order to explore correlations with DTI metrics, we obtained five domain-level subtotals from the SP: Auditory Processing, Visual Processing, Tactile Processing, Multisensory Processing, and Inattention/Distractibility.

Cognitive ability was assessed using the Wechsler Intelligence Scale for Children-Fourth Edition (WISC-IV; Wechsler, 2003). To minimize potential confounding effects of intellectual disability and increase the likelihood of successfully scanning participants without sedation, inclusion was based on a Full-Scale IQ (FSIQ) ≥70. Additional measures of cognitive ability obtained from the WISC-IV include Verbal Comprehension Index (VCI), Perceptual Reasoning Index (PRI), Working Memory Index (WMI), and Processing Speed Index (PSI).

Finally, the Differential Screening Test for Processing (DSTP; Richard and Ferre, 2006), a direct assessment used to identify problems with auditory processing at three levels—acoustic, acoustic linguistic, and linguistic—was administered to all subjects. The acoustic subtest assesses auditory processing skills not associated with language, including binaural integration of two different sets of numbers, recognition of patterns between musical tones, and discrimination of non-sense syllables against a background of white noise. The acoustic linguistic subtest assesses auditory processing associated with language and includes phonemic and phonic manipulation tasks. The linguistic subtest assesses understanding of semantics and pragmatics of language, such as knowledge of antonyms, prosodic interpretation, and the ability to guess a word based on descriptive clues. An overall DSTP Total Score was also calculated by totaling the three subtest scores. Because DSTP scores provide more objective measures of the specific aspects of auditory processing than the SP Auditory score, DSTP scores were also included in correlational analyses with DTI metrics.

### Image Acquisition Protocol

Brain scans were performed using a 12-channel head coil on a 3-Tesla MRI scanner (Siemens Tim Trio; Erlangen, Germany). Whole-brain DTI scans were obtained using a diffusion-weighted echoplanar sequence with echo time = 8,000 ms; repetition time = 109 ms, field of view = 220 mm; voxel size = 2.2 × 2.2 × 2.2 mm; 64 diffusion directions at b-value of 2,000 s/mm^2^; and one brain volume at b-value of 0 s/mm^2^. T1-weighted images were acquired using 3-dimensional magnetization-prepared rapid acquisition gradient echo for anatomical registration (echo time = 2.98 ms, repetition time = 2,300 ms, inversion time = 900 ms, flip angle = 9°). Scan time totaled 60 min per participant.

### DTI Image Processing

The FMRIB Software Library (FSL) version 5.0.8 (Oxford, UK) software was used for image processing. All steps of the image processing pipeline have been reported previously (Owen et al., 2013; Chang et al., 2016; Payabvash et al., 2019). For each subject, the diffusion MRI data were corrected for eddy distortions and motion using FSL eddy_correct. All diffusion-weighted volumes were registered to the reference b = 0 s/mm^2^ volume. To evaluate subject motion, relative displacement was quantified by calculating the transformation of each diffusion volume to the reference. Included subjects were verified to be free of image artifacts and excess movement, defined as more than 2 mm of translation and/or rotation. A heteroscedastic two-sample Student's *t-*test verified that there were no significant differences between SPD and TDC groups in movement during the DTI scan (*p* > 0.05) The registered images were skull-stripped using the Brain Extraction Tool (BET) from FSL. The Diffusion Toolbox in FSL (DTIFIT) was applied on corrected diffusion scans to calculate FA, MD, RD, and AD maps.

Tract-Based Spatial Statistics (TBSS) in FSL (Smith et al., 2006) was used to skeletonize and register the diffusion maps for each subject in order to perform region of interest (ROI) measurements along the white matter skeleton using the Johns Hopkins University (JHU) ICBM-DTI-81 White-Matter Labeled Atlas (Oishi et al., 2009). This is a fully automated process that does not require any manual measurements. Using TBSS, “the most representative subject” was determined from the FA maps of all participants and used as the target image, as recommended for populations of young children. The target image was affine-aligned into MNI152 standard space. Each FA map is transformed by combining the non-linear transform to the target FA image and the affine transform from the determined target image to MNI152 space. The registered FA maps were then averaged and thinned to generate a mean FA skeleton to represent the center of all white matter tracts. The FA white matter skeleton was thresholded to FA > 0.2 to exclude voxels containing gray matter and partial volume effects. Next, each subject's FA data was projected onto this mean skeleton to get individual skeletonized FA maps. The skeleton voxels were filled with values from the nearest relevant tract center by searching perpendicular to the local skeleton structure for the maximum value in the FA image of the subject. Each subject's MD, AD, and RD maps were then registered and projected onto the white matter skeleton.

Five WM tracts were examined: the JHU ICBM-DTI-81 WM Atlas tracts of the SCP-L and SCP-R, the MCP, and the CP-L and CP-R. The JHU Atlas ROIs are defined on the WM skeleton in MNI152 space that is the output of the TBSS pipeline (Figure 1). The SCP, which contains WM fiber tracts that connect the cerebellum with the midbrain, and the MCP, which contains WM fiber tracts that originate in the pontine nuclei, were examined as they are the only outflow tract from the cerebellum and the largest inflow tract to the cerebellum, respectively. The CP was chosen as a control region, since it contains cerebral tracts and has not been previously studied in SPD. The inferior cerebellar peduncles were not analyzed due to their small size and location near the skull base. FA, MD, AD, and RD values of the WM regions were calculated by averaging the voxels within the tract ROI mask for each subject.

### Statistical Analyses

All statistical analyses were conducted using SPSS statistical software. Group characteristics were compared using Student's *t-*tests for continuous and Fisher's exact-tests for nominal variables. Significance was determined at a 95% confidence interval. Two-tailed *t-*tests were run to determine group differences in mean AD, FA, MD, and RD values in the SCP-L and -R, MCP, and CP-L and -R tracts. Pearson correlations of FA, MD, and RD with the WISC-IV components (PRI, WMI, PSI, and FSIQ), DSTP components (acoustic, acoustic linguistic, and total score), and the SP subtests (auditory, visual, tactile, attention, and multisensory processing) in the five examined tracts were investigated by each group separately. Individual one-way ANOVAs were conducted to determine group interaction effects of significant correlations. Multiple comparison corrections were not performed because a prior hypothesis existed for each statistical test performed. Additional *post-hoc t-*tests were run to assess differences in mean AD, FA, MD, and RD values in the SCP-L and -R, MCP, and CP-L and -R tracts between the subset of the SPD cohort with ADHD and the subset of the SPD cohort without ADHD.

## Results

### Participant Characteristics

A total of 42 children with SPD (13 female), and 39 TDC (8 female) were included in our analyses. Table 1 provides their demographic and psychometric characteristics. Statistical analyses showed no significant differences between the SPD and TDC cohorts with regard to gender, age, or general cognitive performance. The cohorts did show significantly different PSI scores from the WISC-IV (*p* = 0.02).

**Table 1:** Subject characteristics.

	**SPD (*n =* 42)**	**TDC (*n =* 39)**	***P*-value**
Age (years)	9.85 (1.33)	10.20 (1.08)	0.207
Sex (female)	13/42 (31%)	8/39 (21%)	0.320
VCI	117.19 (13.99)	121.95 (12.57)	0.118
PRI	112.17 (14.33)	113.47 (12.63)	0.670
WMI	108.05 (14.15)	110.00 (10.77)	0.493
PSI	93.79 (11.99)	100.68 (13.39)	**0.020**
FSIQ	111.88 (12.59)	116.21 (10.24)	0.097

*The first two columns provide group-level means with standard deviations in parentheses for age, verbal comprehension index (VCI), perceptual reasoning index (PRI), working memory index (WMI), processing speed index (PSI), and full scale IQ (FSIQ). For sex, the values listed in the first two columns are number of females per group, with percentage of females in parentheses. The third column lists p-values from group comparisons, with significant values at p < 0.05 bolded*.

### DTI Group Differences Between SPD and TDC

**Figure 2:**
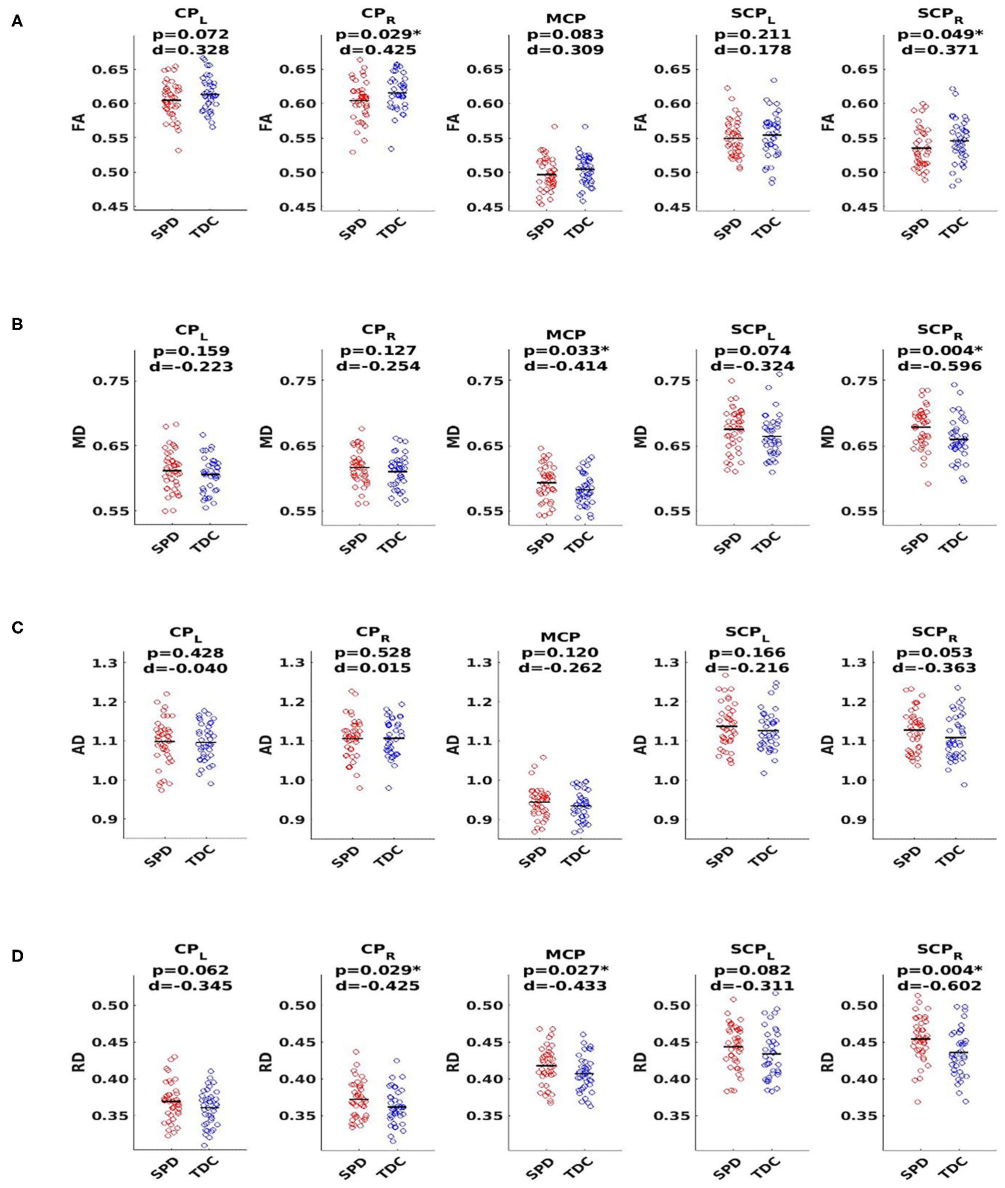
DTI group comparison of typically developing controls (TDC, blue) to sensory processing dysfunction subjects (SPD, red) using fractional anisotropy (FA), mean diffusivity (MD), axial diffusivity (AD), and radial diffusivity (RD). Tracts include the cerebral peduncles (CP, right and left), middle cerebellar peduncle (MCP), and superior cerebellar peduncles (SCP, right and left). **(A)** FA shows significant differences for CP-R and SCP-R, with lower FA in SPD subjects. **(B)** MD plots show significant differences for MCP and SCP-R, with higher MD in SPD subjects. **(C)** AD plots show no significant differences for any of the five white matter tracts examined. **(D)** RD plots show significant differences for CP-R, MCP, and SCP-R with higher RD in SPD subjects. Statistical significance at *p* < 0.05 is indicated by an asterisk. *d* Represents Cohen's *d* effect size.

Using a threshold *p-*value of 0.05, analyses shown in Figure 2 revealed significant differences between SPD and TDC groups in the SCP-R for MD, RD, and FA. There were no significant differences in the SCP-L; however, strong trends in the hypothesized direction were observed. The MCP showed significant group differences for MD and RD. The CP-R showed significant differences for FA and RD. Although not significant, trends in the hypothesized direction were seen for the CP-L. There were no significant group differences in AD in any of the white matter tracts examined. As expected, the SPD group showed lower FA and higher MD and RD values compared to the TDC group in all tracts with significant group differences in DTI metrics.

When dividing the SPD group into ADHD-positive and ADHD-negative subgroups, *t-*tests revealed no significant group differences in any of the tracts examined for AD, MD, RD, or FA.

### DTI Correlations With Direct Assessment of Sensory Measures

**Figure 3:**
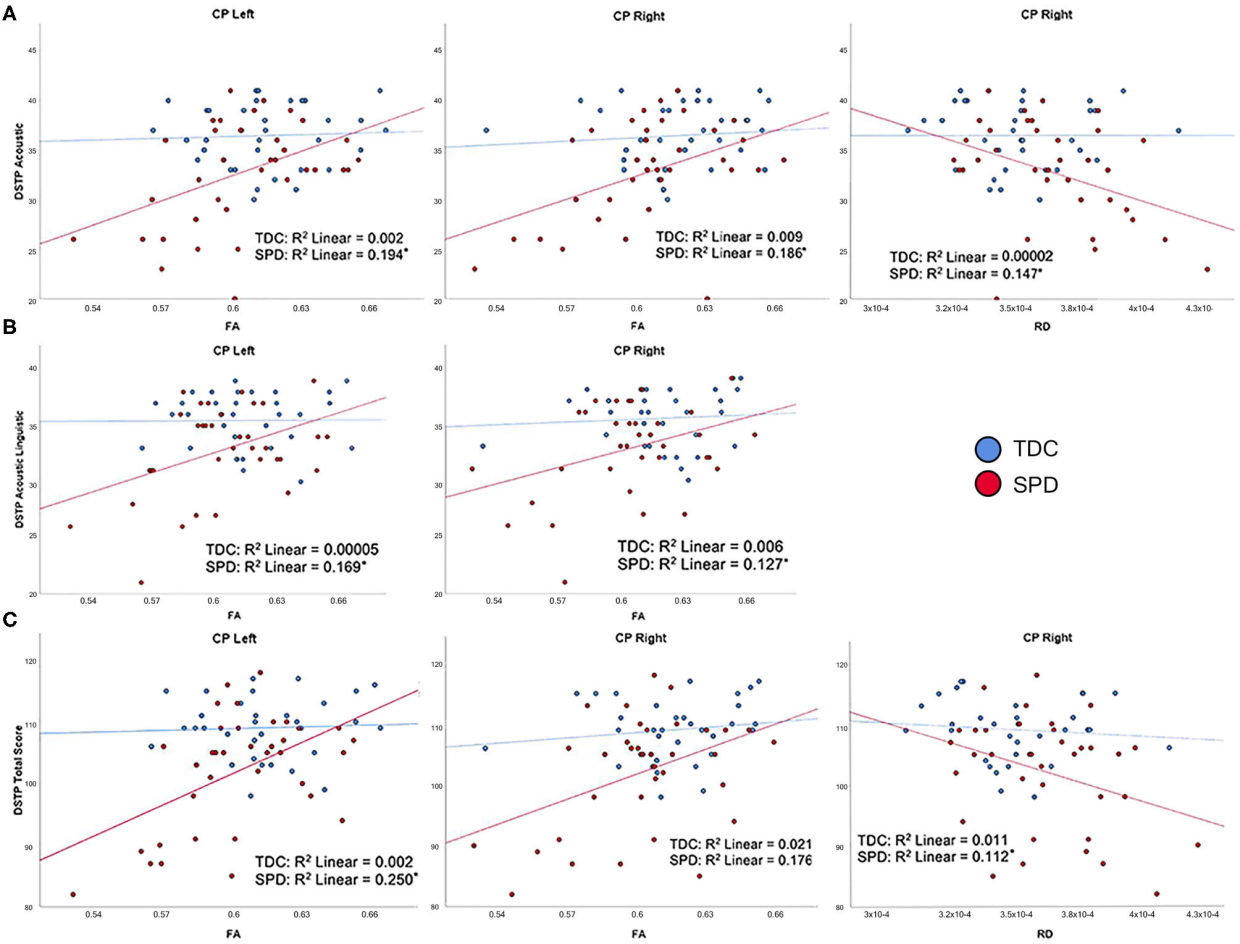
Correlation of TDC (blue) and SPD subjects (red) with Differential Screening Test for Processing (DSTP) scores. Statistical significance at *p* < 0.05 is denoted with an asterisk next to the R^2^ value. **(A)** DSTP acoustic subtest score is correlated with FA in CP-L, FA in CP-R, and RD in CP-R for SPD but not TDC subjects. **(B)** DSTP acoustic linguistic subtest score is correlated with FA in CP-L and FA in CP-R for SPD but not TDC subjects. **(C)** DSTP total score is correlated with FA in CP-L, FA in CP-R, and RD in CP-R for SPD but not TDC subjects.

On the DSTP, a direct assessment of auditory discrimination, acoustic, acoustic linguistic, and DSTP total scores were significantly correlated with FA and RD in the CP-L and CP-R tracts in the SPD group but not the TDC group, consistent with our hypotheses (Figure 3). As expected, lower FA and higher RD were both associated with poorer auditory discrimination performance. However, no significant correlations were found between any of the DTI metrics examined and the DSTP linguistic score in either the SPD or TDC groups.

### DTI Correlations With Parental Assessment of Unimodal Sensory Processing

**Figure 4:**
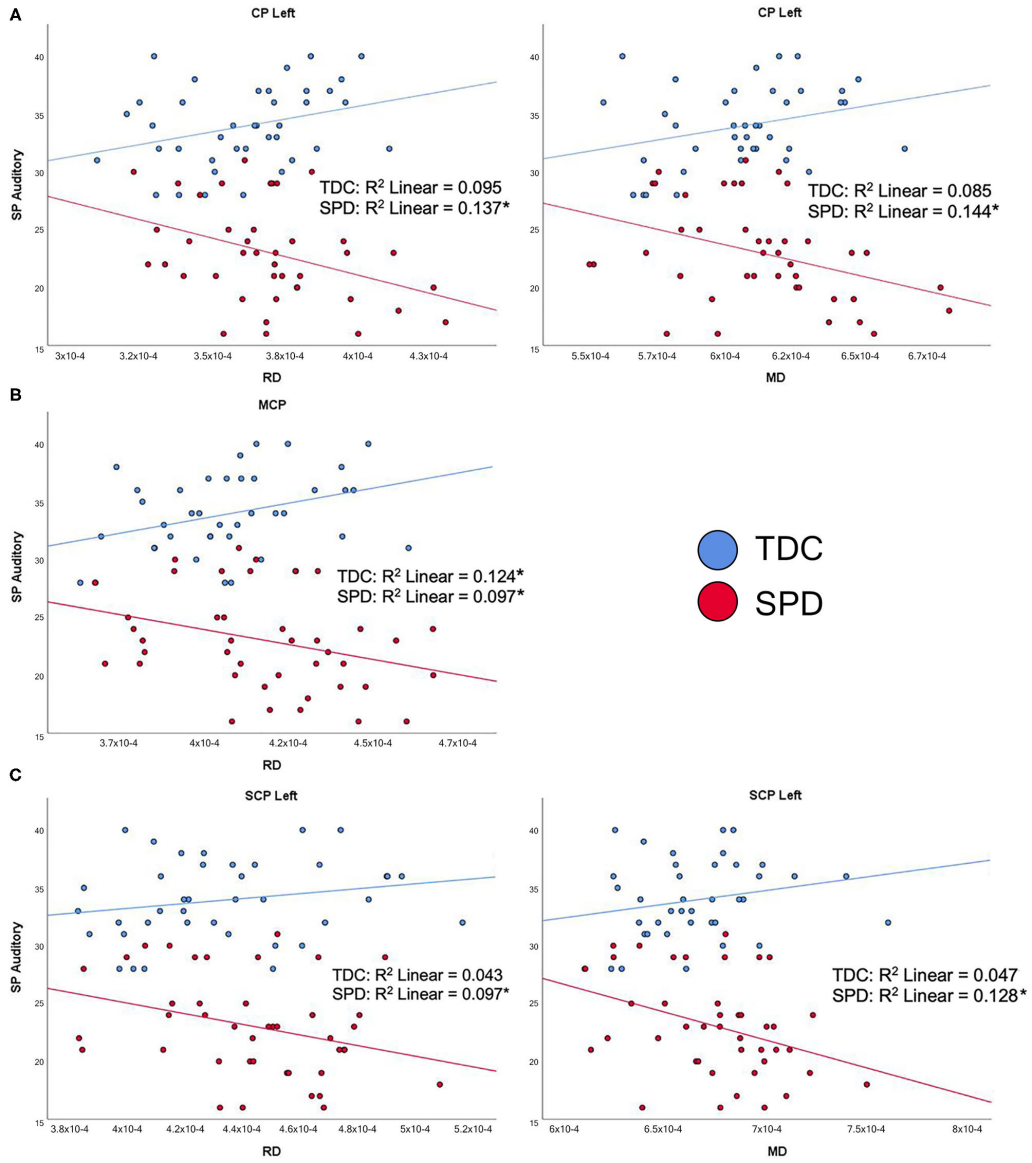
Correlation of TDC (blue) and SPD subjects (red) with the Sensory Profile (SP) auditory processing score. Statistical significance at *p* < 0.05 is denoted with an asterisk next to the R^2^ value. **(A)** SP auditory processing score is correlated with RD in CP-L and MD in CP-L for SPD but not TDC subjects. **(B)** SP auditory processing score is correlated with RD in MCP for both SPD and TDC subjects. **(C)** SP auditory processing score is correlated with RD in SCP-L and MD in SCP-L in SPD but not TDC subjects.

The SP Auditory Processing subtotal significantly correlated with RD and MD values of all five tracts examined, which include both cerebellar and cerebral peduncles, in the SPD group (Figure 4). Additionally, there were significant group by tract interaction effects for RD and MD in the CP-L, CP-R, MCP, and SCP-L (Table 2). These results were consistent with the hypothesized relationship between higher diffusivities and more impaired sensory processing. Few significant correlations were observed in the TDC group, including RD in the MCP, where the correlation was reversed compared to the SPD group (Figure 4). Correlations that were significant for the TDC but not the SPD group included MD and RD in the CP-R and FA in the MCP. These correlations showed a similar pattern to RD in the MCP, where higher diffusivity correlated with less impaired sensory processing for TDC, the opposite relationship expected in the SPD group. This disparity in the correlation of DTI metrics with the SP Auditory Processing measure between the SPD and TDC groups is shown to be statistically significant as a group by tract interaction effect (Table 2).

**Table 2:** Group, tract, and interaction effects of Sensory Profile scores and DTI metrics.

**Sensory Profile**											
**Metric**	**Tract**	**Inattention**			**Auditory processing**			**Tactile processing**			**Multisensory processing**		
		**Effect of cohort**	**Effect of tract**	**Interaction effect**	**Effect of cohort**	**Effect of tract**	**Interaction effect**	**Effect of cohort**	**Effect of tract**	**Interaction effect**	**Effect of cohort**	**Effect of tract**	**Interaction effect**
FA	CP-L	0.055	0.922	0.152	**0.025**	0.993	0.080	**0.003**	0.102	**0.010**	0.208	0.659	0.383
	CP-R	**0.014**	0.788	0.051	**0.016**	0.617	0.058	**0.020**	0.358	0.059	0.209	0.680	0.393
	MCP	**0.001**	0.480	**0.004**	**0.002**	0.618	**0.008**	0.129	0.675	0.296	0.075	0.881	0.171
	SCP-L	**0.002**	0.169	**0.010**	0.074	0.994	0.245	0.127	0.353	0.329	**0.005**	0.095	**0.019**
	SCP-R	**0.003**	0.453	**0.021**	0.070	0.800	0.248	**0.024**	0.130	0.091	**0.008**	0.229	**0.029**
MD	CP-L	0.244	0.769	0.086	**0.016**	0.612	**0.003**	0.822	0.281	0.469	0.407	0.885	0.208
	CP-R	0.157	0.794	0.055	**0.017**	0.563	**0.004**	0.708	0.656	0.941	0.664	0.596	0.409
	MCP	0.204	0.286	0.076	**0.049**	0.785	**0.013**	0.769	0.969	0.870	0.408	0.450	0.224
	SCP-L	0.095	0.070	**0.025**	**0.040**	0.359	**0.009**	0.765	0.366	0.420	0.288	0.201	0.134
	SCP-R	0.321	0.167	0.124	0.354	0.639	0.132	0.644	0.265	0.340	0.528	0.198	0.293
RD	CP-L	0.209	0.752	**0.039**	**0.025**	0.566	**0.002**	0.169	0.077	**0.035**	0.399	0.969	0.141
	CP-R	0.073	0.800	**0.010**	**0.027**	0.517	**0.003**	0.521	0.354	0.182	0.540	0.620	0.220
	MCP	0.061	0.255	**0.010**	**0.028**	0.992	**0.004**	0.951	0.876	0.562	0.371	0.624	0.149
	SCP-L	**0.024**	**0.028**	**0.002**	0.124	0.371	**0.016**	0.780	0.259	0.297	0.066	**0.048**	**0.013**
	SCP-R	0.113	0.153	**0.017**	0.449	0.753	0.110	0.379	0.100	0.111	0.156	0.092	**0.042**

*P-values from one-way ANOVAs examining main effects of group, main effects of tract, and interaction effects for inattention, auditory, tactile, and multisensory processing subtotals from the SP. Significant values at p < 0.05 are in bold*.

**Figure 5:**
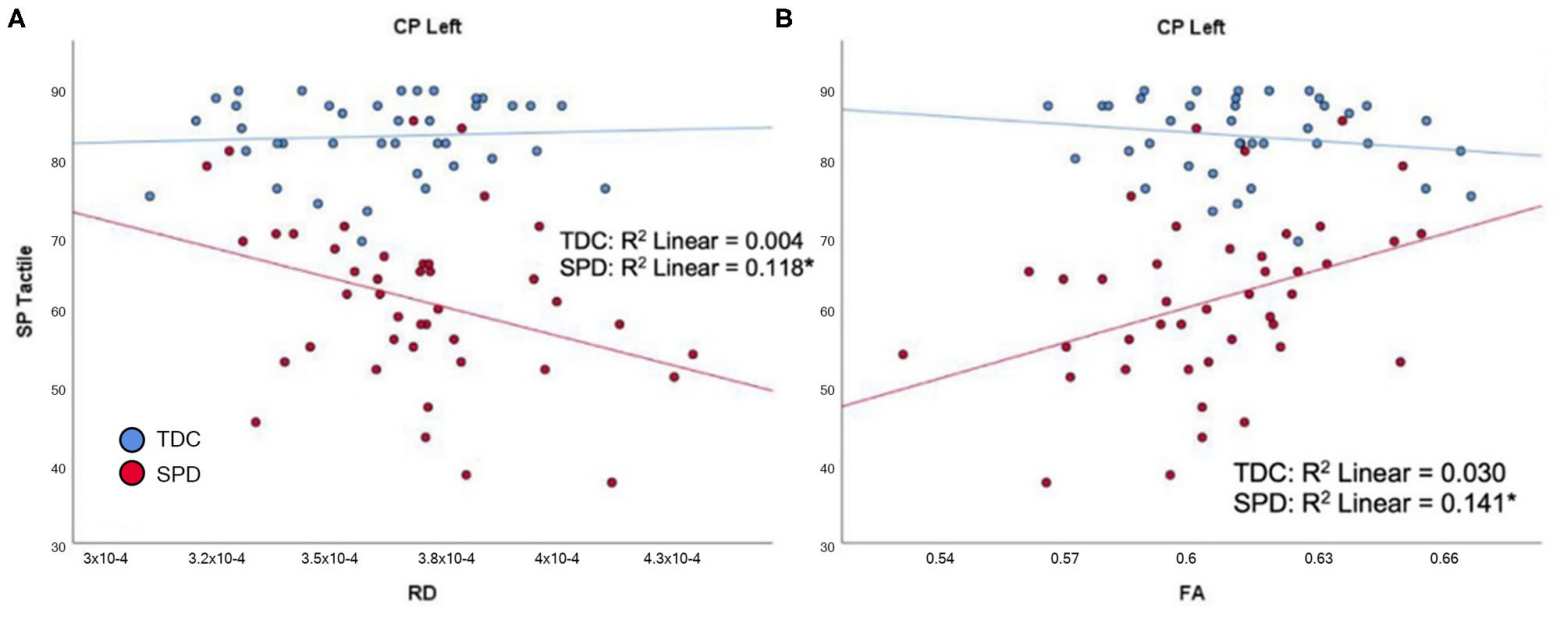
Correlation of TDC (blue) and SPD subjects (red) with SP tactile processing score. Statistical significance at *p* < 0.05 is denoted with an asterisk next to the R^2^ value. **(A)** SP tactile processing score is correlated with RD in CP-L for SPD but not TDC subjects. **(B)** SP tactile processing score is correlated with FA in CP-L for SPD but not TDC subjects.

In the SPD group, the SP Tactile Processing subtotal was significantly correlated with RD and FA of the CP-L (Figure 5), but none of the cerebellar tracts. No significant correlations were found in the TDC group. Therefore, significant group by tract interaction effects in tactile processing were identified for RD and FA of the CP-L (Table 2).

### DTI Correlations With Parental Assessment of Multisensory Integration and Attention

**Figure 6:**
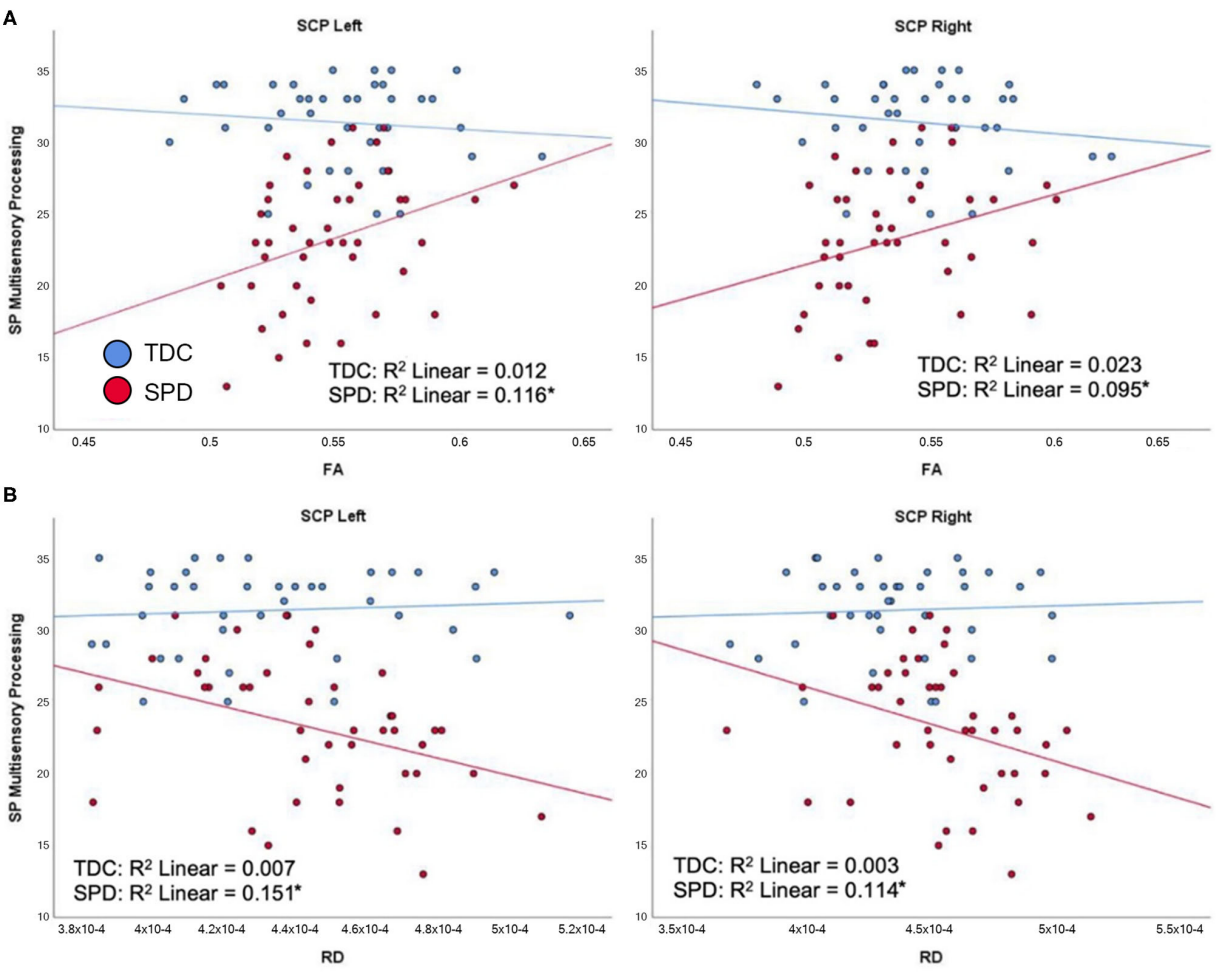
Correlation of TDC (blue) and SPD subjects (red) with SP multisensory processing score. Statistical significance at *p* < 0.05 is denoted with an asterisk next to the R^2^ value. **(A)** SP multisensory processing score is correlated with FA in SCP-L and FA in SCP-R for SPD but not TDC subjects. **(B)** SP multisensory processing score is correlated with RD in SCP-L and RD in SCP-R for SPD but not TDC subjects.

**Figure 7:**
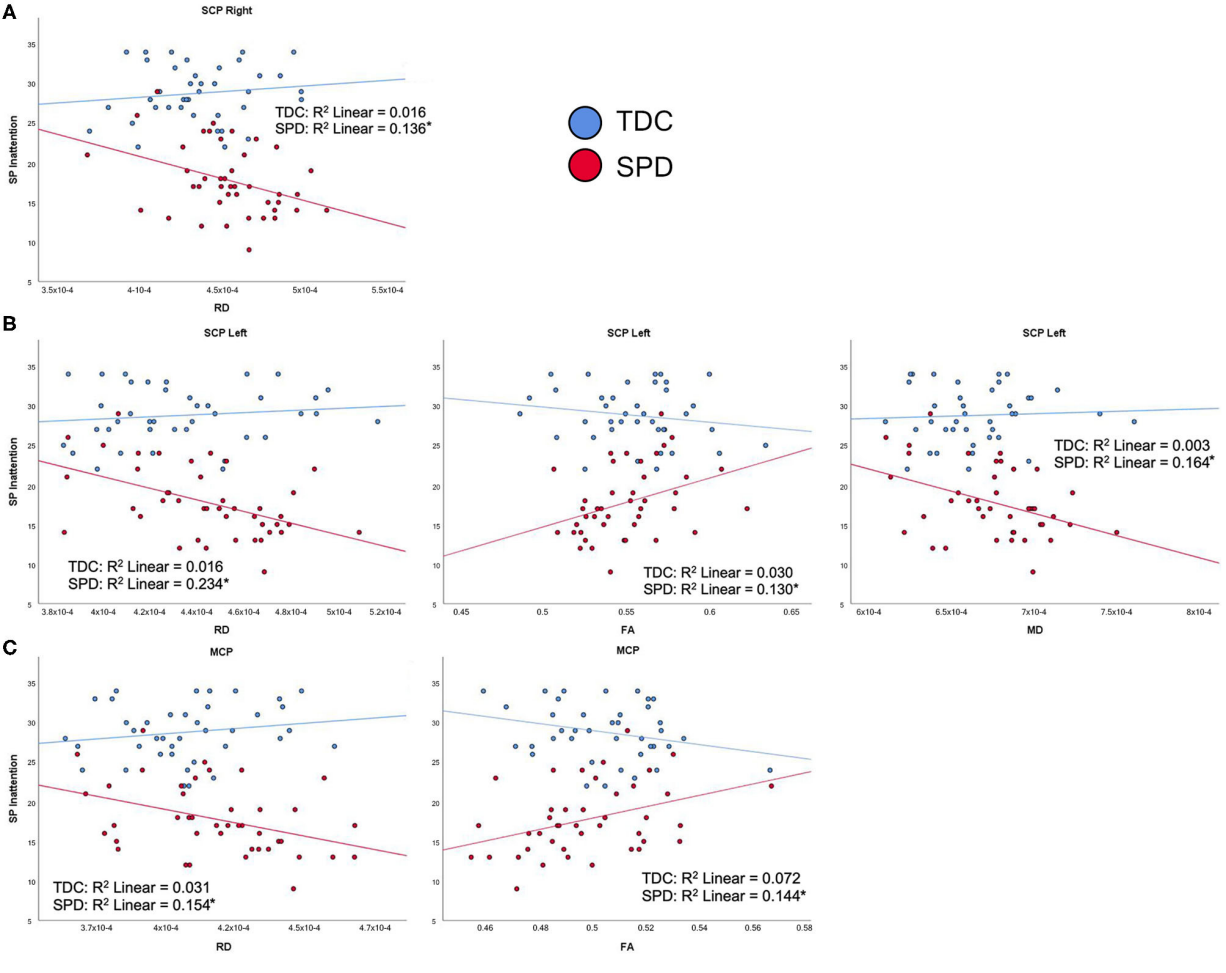
Correlation of TDC (blue) and SPD subjects (red) with SP inattention score. Statistical significance at *p* < 0.05 is denoted with an asterisk next to the R^2^ value. **(A)** Sensory Profile (SP) inattention score is correlated with RD in SCP-R for SPD but not TDC subjects. **(B)** SP inattention score is correlated with RD in SCP-L, FA in SCP-L, and MD in SCP-L for SPD but not TDC subjects. **(C)** SP inattention score is correlated with RD in MCP and FA in MCP for SPD but not TDC subjects.

For the SPD group, the SP Multisensory Processing subtotal was significantly correlated with RD and FA in the SCP-L and SCP-R (Figure 6). No significant correlations were found for the TDC group; hence, significant group by tract interaction effects were found for RD and FA of the SCP-L and SCP-R (Table 2). The SP Inattention subtotal was significantly correlated with RD, MD, and FA of the SCP-L and -R and the MCP in the SPD cohort, but not the TDC cohort (Figure 7). Significant group by tract interaction effects were therefore identified for RD and FA of all the cerebellar tracts (Table 2). Neither the SP Multisensory Processing nor the SP Inattention subtests showed any significant correlation with DTI metrics of the left or right cerebral peduncles for the SPD group, though the TDC group showed a significant positive correlation between RD in the CP-R and Inattention score. Once again, the relationship seen in the TDC group is opposite what we would expect to see in the SPD group.

## Discussion

### Alterations of Brainstem and Cerebellar White Matter Microstructure in SPD

Our results demonstrate that children with SPD have altered brainstem and cerebellar WM microstructural integrity relative to TDC. As hypothesized based on our previous studies of the cerebral hemispheres (Owen et al., 2013; Chang et al., 2016; Payabvash et al., 2019), these microstructural changes of the cerebral and cerebellar peduncles consist of reduced FA, increased MD, and increased RD. Consistent with these prior reports, RD typically showed the largest effect sizes, followed by FA and MD, whereas AD had the weakest effects. This supports the underlying biological principle that barriers to microscopic water diffusion radial to the orientation of axonal fiber bundles are not as well-developed in SPD as they are for TDC. While recent studies have shown altered cerebellar connectivity in individuals with ASD (Cardon et al., 2017; Oldehinkel et al., 2019), none have explored this relationship in children with SPD who do not have additional social-communication deficits meeting criteria for ASD. By examining an SPD cohort, we are able to investigate sensory processing dysfunction more directly, without the confounding effect of the additional brain-based cognitive and affective challenges. The fact that significant differences in cerebellar white matter microstructure exist between the two groups when isolating sensory dysfunction adds to increasing evidence that the cerebellum plays an important role in sensory processing and attention. Furthermore, evidence from a mouse model of white matter injury of prematurity shows a loss of GABA_A_ receptor–mediated synaptic input to cerebellar NG2 cells, resulting in extensive proliferation of these cells and delayed oligodendrocyte maturation, eventually leading to dysmyelination (Zonouzi et al., 2015). Prematurely born children demonstrate elevated rates of SPD (Wickremasinghe et al., 2013) and are known to have WM diffusivity changes on DTI that are characteristic of diminished myelination (i.e., elevated RD and MD as well as reduced FA) similar to those we demonstrate herein for the cerebellar and cerebral peduncles in term-born children with SPD. A new study of neurotypical children has shown a strong correlation between FA in the right and left SCP and quantitative T1 values, a more specific measure of myelination than DTI metrics, providing further support that our findings are at least in part due to myelination differences between groups (Bruckert et al., 2020). Thus, abnormal excitatory-inhibitory balance during cerebellar development may represent a possible mechanism for producing these microstructural WM alterations that are ultimately expressed as the SPD phenotype.

### Cerebral vs. Cerebellar Peduncle Microstructure and Unimodal Sensory Processing

Also consistent with our earlier findings about white matter of the cerebral hemispheres in SPD (Chang et al., 2016), WM microstructure of the left and right CPs in the SPD children was significantly correlated with all but the linguistic subtest scores of the DSTP, which is a direct assessment of auditory processing. In contradistinction, the SCPs and MCP in the SPD group were not associated with any of the DSTP scores, indicating that cerebellar white matter is not as directly involved in impaired auditory discrimination as is cerebral white matter.

Similar to the DSTP measures, the SP Tactile subtotal was associated with cerebral peduncle microstructure but not cerebellar peduncle microstructure. However, the SP Auditory subtotal was significantly correlated with WM microstructure in *both* cerebellar and cerebral peduncles. One possible explanation for this discrepancy is that the SP combines all aspects of auditory processing, including modulation, discrimination, and sensory-based motor issues, into a single auditory processing score, while the DSTP examines auditory discrimination specifically. However, Parsons et al. (2009) showed a relationship between impaired cerebellar functioning and poor performance on a pitch discrimination task, which does support the idea that the cerebellum may be important in some aspects of auditory discrimination. Another explanation is that certain questions in the SP involve both auditory and attention elements, such as “Is distracted or has trouble functioning if there is a lot of noise around,” which makes it difficult to assess auditory processing and attention separately using only the SP. Finally, these results may reflect the fact that the SP is a subjective parent report measure involving a behavioral questionnaire and the DSTP is a more objective, direct assessment. Previous research has shown that direct assessment of sensory processing is more strongly correlated with measures of cerebral WM microstructure than are parent report measures, which could potentially be due to parental bias (Chang et al., 2016).

RD showed the most consistent relationships with DSTP as well as SP Auditory and Tactile measures in the SPD cohort, followed by FA and MD. As expected, AD did not show any significant associations with any of the sensory measures in either the cerebral or cerebellar peduncles. Surprisingly, the reverse correlation between the SP Auditory measure and RD of the MCP was observed for TDC compared to the SPD children, with trends toward reversed correlations in the left and right SCP as well. More data is needed to verify these results in the TDC group that run counter to the hypothesized relationship between white matter microstructure and sensory processing. In general, the TDC group did not show significant correlations between cerebral or cerebellar peduncle WM microstructure and sensory behavior, even those with RD, FA and MD values in the same range as most SPD kids. This suggests that typically developing children are often more resilient to the potential effects of altered cerebellar white matter microstructure, perhaps because of other compensatory neural pathways. This remains an area for further exploration.

### Cerebellar White Matter Microstructure and Higher-Order Sensory Behavior

Although the cerebellum has historically been implicated primarily in motor coordination, it is now clear that the cerebellum and its cortical connections are also important for higher-order processes such as multisensory integration, perceptual analysis, working memory, attention, and social cognition (Baumann et al., 2015; Sathyanesan et al., 2019; Van Overwalle et al., 2020). As we hypothesized, significant correlations exist between white matter microstructure of the cerebellar peduncles and parent report measures of inattention and multisensory processing in the SPD cohort. The SCP, which we found to be associated with both multisensory integration and inattention SP measures, contains the cerebellothalamic tract, which transmits information via the thalamus to cerebral cortex, including higher-order association areas (Martin, 2003; Buckner, 2013). The MCP, which along with the SCP was associated with inattention measures in our study, receives connections from these same areas of cerebral cortex via the pons (Wang et al., 2003; Buckner, 2013). Integration of auditory, somatosensory, and visual information is known to occur via mossy fibers that synapse onto cerebellar granule cells in animal experiments (Groen et al., 2011; Huang et al., 2013). While a few previous studies in humans have suggested that the cerebellum may facilitate multisensory integration (Kern, 2002; Erickson et al., 2014), they have been limited to small sample sizes, including a case report of cerebellar agenesis (Ronconi et al., 2017). The current study provides additional clinical evidence that the cerebellum is involved in dysfunctional multisensory integration. Moreover, it is well-established from human resting state fMRI studies that the cerebellum is an integral part of both the dorsal and ventral attention networks (Buckner et al., 2011). Our results corroborate this finding by showing altered cerebellar microstructural WM organization is related to abnormal attentional control in a clinical phenotype characterized by sensory processing dysfunction.

### Limitations and Future Studies

As an initial exploration of the role of cerebellar WM microstructure in sensory processing dysfunction, this study has several limitations. To more comprehensively determine how altered WM microstructure affects sensory behavior, future studies should include a full battery of both parent report and direct observation measures. The inclusion of more objective child assessments eliminates parental bias and provides insight into which parent report measures are most reflective of directly observable sensory function. Additionally, further investigations with larger sample sizes can focus on analyzing SPD using more homogeneous subcategories based on sensory domain (e.g., tactile, auditory) and/or type of processing issue (e.g., over- or under-responsivity, seeking, discrimination). With an SPD sample as defined in the current study, it is possible to have a cohort of individuals with “opposite” sensory symptoms (e.g., sensory over- vs. under-responsivity) that might obscure important findings that could be made in each distinct subgroup. Another limitation is the inclusion of children with and without ADHD in the SPD cohort. Given our results, which suggest the cerebellum plays a role in attention in children with SPD, it would be useful to assess SPD and ADHD separately in future studies. However, a *post-hoc* analysis showed no significant difference in cerebellar or brainstem DTI metrics in SPD participants who screened positive for ADHD vs. those who screened negative, indicating that ADHD was not the primary cause of the altered microstructural integrity found in this study. The current study was also limited in terms of certain aspects of generalizability. For example, our subjects were chosen to fall within a narrow age range prior to puberty to reduce variability in this moderate-sized cohort as well as to avoid the challenges of scanning unsedated younger children. Future longitudinal studies should include a wider age range to ensure generalizability and determine how WM integrity in SPD is affected over the course of neurodevelopment. These investigations could also use new diffusion MRI methodology such as neurite orientation dispersion and density imaging (NODDI) for more sensitive and specific evaluation of white matter microstructure (Zhang et al., 2012; Owen et al., 2014; Chang et al., 2015), including assessing children before and after various treatments, such as occupational therapy.

### Conclusions

In summary, the current study validates previous findings of altered WM connectivity in children with SPD compared to TDC. It also demonstrates the cerebellum's role in multisensory integration and attention in individuals with SPD. Finally, the current study contributes to the overall understanding of the neuroanatomical features present in SPD. It is important to identify these neurobiological underpinnings of the disorder, which can ultimately provide a biomarker to inform classification and diagnosis, prognostication, and interventions. It may also lend further support to SPD being considered a “brain-based” condition, rather than as a secondary symptom of ASD or a result of poor parenting, and help those with SPD obtain the knowledge and treatment options they need.

## Data Availability Statement

The datasets presented in this study can be found in online repositories. The names of the repository/repositories and accession number(s) can be found at: https://datadryad.org/stash/share/nFhEh3cIfo635GI_yE3xWcsf40QC_q1s1lSVDubZVwA Repository: Dryad Name: Altered cerebellar white matter in sensory processing dysfunction is associated with impaired multisensory integration and attention.

## Ethics Statement

The studies involving human participants were reviewed and approved by University of California, San Francisco Institutional Review Board. Written informed consent to participate in this study was provided by the participants' legal guardian/next of kin.

## Author Contributions

EM and PM: conception and design of the study. AN, MR, EP, JW-J, IB, MG, AB-A, and SD: acquisition and analysis of the data. AN, MR, EP, EM, and PM: drafting significant portion of the manuscript and figures. All authors contributed to the article and approved the submitted version.

## Conflict of Interest

MR, MG, and EM were employed by Cortica Healthcare. The remaining authors declare that the research was conducted in the absence of any commercial or financial relationships that could be construed as a potential conflict of interest.
